# Engaging Gatekeeper-Stakeholders in Development of a Mobile Health Intervention to Improve Medication Adherence Among African American and Pacific Islander Elderly Patients With Hypertension

**DOI:** 10.2196/mhealth.5905

**Published:** 2016-10-26

**Authors:** Hamed Yazdanshenas, Mohsen Bazargan, Loretta Jones, May Vawer, Todd B Seto, Summer Farooq, Deborah A Taira

**Affiliations:** ^1^College of MedicineDepartments of Family Medicine and Orthopedic SurgeryCharles R Drew University of Medicine and Science/University of California, Los Angeles (UCLA)Los Angeles, CAUnited States; ^2^College of MedicineDepartment of Family MedicineCharles R Drew University of Medicine and Science/University of California, Los Angeles (UCLA)Los Angeles, CAUnited States; ^3^Department of Community EngagementCharles R Drew University of Medicine and ScienceLos Angeles, CAUnited States; ^4^The Queen's Medical Center and the John A Burns School of Medicine, University of HawaiiHonolulu, HIUnited States; ^5^College of MedicineDepartment of Family MedicineCharles R Drew University of Medicine and ScienceLos Angeles, CAUnited States; ^6^Daniel K Inouye College of PharmacyUniversity of Hawai‘i at HiloHonolulu, HIUnited States

**Keywords:** health, technology, elderly, minority, hypertension, mHealth

## Abstract

**Background:**

Approximately 70 million people in the United States have hypertension. Although antihypertensive therapy can reduce the morbidity and mortality associated with hypertension, often patients do not take their medication as prescribed.

**Objective:**

The goal of this study was to better understand issues affecting the acceptability and usability of mobile health technology (mHealth) to improve medication adherence for elderly African American and Native Hawaiian and Pacific Islander patients with hypertension.

**Methods:**

In-depth interviews were conducted with 20 gatekeeper-stakeholders using targeted open-ended questions. Interviews were deidentified, transcribed, organized, and coded manually by two independent coders. Analysis of patient interviews used largely a deductive approach because the targeted open-ended interview questions were designed to explore issues specific to the design and acceptability of a mHealth intervention for seniors.

**Results:**

A number of similar themes regarding elements of a successful intervention emerged from our two groups of African American and Native Hawaiian and Pacific Islander gatekeeper-stakeholders. First was the need to teach participants both about the importance of adherence to antihypertensive medications. Second, was the use of mobile phones for messaging and patients need to be able to access ongoing technical support. Third, messaging needs to be short and simple, but personalized, and to come from someone the participant trusts and with whom they have a connection. There were some differences between groups. For instance, there was a strong sentiment among the African American group that the church be involved and that the intervention begin with group workshops, whereas the Native Hawaiian and Pacific Islander group seemed to believe that the teaching could occur on a one-to-one basis with the health care provider.

**Conclusions:**

Information from our gatekeeper-stakeholder (key informant) interviews suggests that the design of a mHealth intervention to improve adherence to antihypertensives among the elderly could be very similar for African Americans and Native Hawaiian and Pacific Islanders. The main difference might be in the way in which the program is initiated (possibly through church-based workshops for African Americans and by individual providers for Native Hawaiian and Pacific Islanders). Another difference might be who sends the messages with African Americans wanting someone outside the health care system, but Native Hawaiian and Pacific Islanders preferring a provider.

## Introduction

Hypertension is the most common condition seen in primary care and may lead to myocardial infarction, stroke, renal failure, and death if not treated appropriately. In 2011, the cost burden in the United States associated with hypertension was estimated at US $46 billion in health care services, medications, and missed days of work [1]. Although advances in pharmacotherapy have decreased morbidity and improved life expectancy, poor medication adherence has offset some of these gains.

Racial and ethnic minority populations bear a disproportionate burden of hypertension and its sequelae. The prevalence of high blood pressure in African Americans is the highest in the world with approximately 32% of African Americans having hypertension. Estimates from the National Health Interview Survey in 2007 found that Native Hawaiians and Pacific Islanders had the second highest rates of any ethnic group in the United States at 29% [2-5]. Despite evidence that appropriate pharmacological treatment reduces morbidity and mortality across all populations [6-8], racial and ethnic minorities, including African Americans and Native Hawaiian and Pacific Islanders, often underutilize antihypertensive medications [9-11].

Even among people who see their providers regularly, 50% are nonadherent to prescribed medications after 6 months [12]. This nonadherence results in thousands of premature deaths, hospitalizations, and increased health care costs [13,14]. Of all medication-related hospital admissions, 33% to 69% are due to poor medication adherence, with approximate health care costs of US $100 billion annually [15,16]. Greater adherence to medications for chronic conditions including hypertension has been associated with higher medication costs, but net overall reduction in total health care costs [17].

Efforts to improve health-related behaviors and provider-patient communication play an increasingly important role in the management of patients with hypertension. Mobile health technology (mHealth) has been shown to be a feasible, well-accepted, and cost-effective means of achieving sustained gains in health-related behaviors, including physical activity, medication adherence, and blood pressure control [18-26]. The use of mHealth offers a platform for innovative approaches to health enhancement and disease management at low cost with widespread applicability, while promoting self-efficacy and autonomous regulation. Importantly, mHealth may be particularly relevant for populations with limited access to health care, including those who live in rural communities, and among racial and ethnic minorities. Examples of mHealth include text-based reminders, electronic delivery of educational materials, and use of mobile devices to monitor physical activity, medication adherence, and blood pressure.

The goal of this study was to better understand issues affecting the acceptability and usability of mobile health technology to improve medication adherence for elderly African American and Native Hawaiian and Pacific Islander patients with hypertension.

## Methods

We conducted semistructured (30 minute) interviews of gatekeeper-stakeholders (N=20) who were health care providers or community leaders and were familiar with issues surrounding medication adherence among elderly African American (n=10) or Native Hawaiian and Pacific Islander (n=10). Potential participants asked to participate in this study were connected to the geriatric population. In addition, attempt was made to invite potential participants who were older. All gatekeeper-stakeholders who were approached agreed to participate. Established qualitative approaches were used to analyze and to interpret data [27,28]. This study was reviewed and approved by the Committee on Human Subjects for the two study sites at The Queen’s Medical Center in Honolulu, HI, and at Charles Drew University in Los Angeles, CA.

### Interviews

Because of the paucity of information about the acceptability of mHealth for African American and Native Hawaiian and Pacific Islander seniors, we chose a qualitative approach to identify themes that could be further explored in future quantitative studies. Two members of the research team conducted in-depth interviews with targeted open-ended questions and follow-up probes to elicit further clarification (Textbox 1). The interview covered the following key topics: (1) major concerns that each ethnic elderly population may encounter related to hypertension medication adherence; (2) ethnic differences, perceptions, and barriers to using mHealth interventions for medication self-management in older adults with hypertension; (3) desired daily frequency of mHealth interventions as well as the assessment of content to ensure cultural and linguistic appropriateness; (4) most effective mode of delivery of mHealth interventions to increase medication adherence; and (5) ways to increase older adults’ interest and awareness about using mHealth interventions to improve compliance with their antihypertensive medications. Gatekeeper-stakeholders received a US $30 gift card for their participation to cover their time.

**Main questions from the semistructured key informant interviews.**

Do you think most seniors in your community have a cell phone? How about a smartphone?

How effective do you think it would be to use a cell phone to remind seniors in your community about taking their blood pressure medication on time?

What would be the best way to phrase the text reminder/question?

From what source do you think seniors in your community would like to receive the reminder to take their blood pressure medication (eg, physician office, nurse, other family members...)?

Whom else do you think should receive the reminders that can help the senior population in your community to take their blood pressure on time?

Are there any cultural considerations?

### Analysis

Interviews were deidentified and transcribed by members of the research team using a combination of direct quotes, paraphrasing, and summarization. They were then organized and coded manually by two independent coders. Analysis of patient interviews used largely a deductive approach because the targeted open-ended interview questions were designed to explore issues specific to the design and acceptability of a mHealth intervention for seniors. We also used inductive methods to identify additional themes that emerged during the interviews. Two primary members of the research team performed the first- and second-level coding. Input from the other members of the team was obtained to confer about the codes, quotes, and interpretation of quotes.

## Results

### Characteristics of Gatekeeper-Stakeholders

A convenience sample of community-based gatekeeper-stakeholders familiar with either the African American (n=10) or Native Hawaiian and Pacific Islander (n=10) elderly community were recruited for the interviews. Overall, the mean age of the gatekeeper-stakeholders (N=20) was 59.4 (SD 13.3) years. All but four participants were 50 years and older. All participants were connected to the geriatric population. The sample included founder or chief executive officer of a nongovernmental organization, community advocates, church leaders, and retired or active health care providers, including physicians, nurses, pharmacist, and medical assistant. Overall, 70% (14/20) of participants were female. All participants had a mobile phone, although one participant had a mobile phone with only a phone feature. All gatekeeper-stakeholders with mobile phones used their phones to access the Internet, but only four said they downloaded apps with their mobile phone.

### Elderly Adults Use of Mobile Phones

When asked if they believe most elderly adults have mobile phones (including smartphones and cell phones), most gatekeeper-stakeholders said that having and using mobile phones by older adults was probably related to age. A common sentiment was that patients older than age 70 or 75 years might not have mobile phones or, if they do, they only use them for emergencies or to talk to relatives:

They just use cellphone for emergency purposes (making and receiving calls), but for other features for using cellphone (like sending text and taking picture), they don’t have interest. The majority of individuals are retirees, age makes a difference; 50-year-olds are more willing to learn all that phones have to offer, once you get to 70, that changes.

P12, African American

A few also mentioned education level of the patient as an indication of whether they would have a mobile phone:

Some of the elderly who are more professional and just retired, they are very savvy with social media and stuff so they are using it in a comprehensive manner. Those who are more progressive and professional, they are texting, Facebooking, Instagramming. Those who maybe don’t have a high school diploma (regular people in the community), worked in blue-collar jobs all their lives, and are just about to retire, they just use it for calling friends and relatives.

P15, African American

The general sentiment among gatekeeper-stakeholders was that lack of a mobile phone would not be a barrier, but that older participants would need a lot of training and support.

### Elements Necessary for Success

There were a number of themes that emerged from both groups and some that arose only from either the African American or the Native Hawaiian and Pacific Islander gatekeeper-stakeholders (Textbox 2). The most frequently mentioned themes for both groups were (1) need to personalize the content; (2) teach about the importance of medication adherence and support people in using their mobile phone; (3) show caring, connection, and trust; (4) keep it simple; and (5) include music. Other suggestions by both groups were to include a picture of the medication pill, to enable people to help one another, and to ask nicely (eg, say “please”).

**Themes for an effective mHealth intervention by group.**

**Both groups**

- Personalized
- Teach, remind, follow up, be available to answer questions
- Caring/love/connection/trust
- Short/simple/easy to understand
- Include music
- Picture of pill
- Enable people to help one another, be contagious
- Ask nicely/say please/include positive affirmations

**African Americans**

- Workshop to teach about mobile phone
- Involve church
- Subsidize/incentivize
- Assistance from family member; voice/photo of family member
- Include quotes from scripture, authors, the President; tips on hobbies; slang/jokes

**Native Hawaiians/Pacific Islanders**

- Cannot be just out of the blue/need plan
- Patient choice/control/involvement/partnership
- Consider where they are at, readiness
- Start by reducing the number of medications if possible

#### Elements Wanted by Both Groups

##### Personalize the Content

Virtually every gatekeeper-stakeholder spoke of the need to personalize the intervention to the individual’s needs:

Whatever can be personalized to the patient the better it will be and at the same time can’t be in isolation, needs to be part of the bigger plan with the doctor-team...

P9, Native Hawaiian/Pacific Islander

##### Teach and Support

Another very common theme was the need to teach, not only about the importance of taking medications as prescribed, but on the use of mobile phones in general. There was also a strong sentiment expressed by many that there would need to be ongoing support. Specific quotes from gatekeeper-stakeholders included:

...Need to talk story about what and why medications being ordered, who to call with questions, follow up to see how it’s going, how they are doing.

P1, Native Hawaiian/Pacific Islander

You can’t give it to them and say “BYE,” then you’ll never hear from them for life. Educate them, follow up, and then follow up after the follow-up.

P14, African American

##### Importance of Caring, Connection, and Trust

Another common theme expressed by gatekeeper-stakeholders in both groups was the need to have the messaging come from someone with whom they have a connection and trust:

A person for them to connect with—so they know them—have worked with, learned what to do—so when they get their texts (to take medications) they know who it’s from [you] it connects them and it’s not just this machine that’s texting them.

P5, Native Hawaiian/Pacific Islander

Love is important. People talk about it with their social groups. Use words like “I care about you” or “I want you to be around” or “we love you.” You can go to Hallmark and see the greeting cards they have. Use a more common, less formal vernacular, like misspelling words. “Wazzup?” Personalize it. Use their names, first name and a picture if possible. Hallmark greeting card language is a suggestion. Maybe using some slang or jokes in the text reminders.

P19, African American

##### Keep it Simple

Regarding the nature of the messaging, the gatekeeper-stakeholders consistently emphasized the need to keep it short and sweet, simple, and direct:

Yeah and not annoying like telemarketing—wasting our time...pause...short and sweet shows a flash of caring.

P4, Native Hawaiian/Pacific Islander

Shorter. People tend to want to read less. People have visual or literacy problems as they get older. Maybe once in a while, use a longer one to break things up (every fifth or tenth text).

P19, African American

##### Include Music

Without prompting, many gatekeeper-stakeholders expressed the idea of including music as part of the messaging:

Yeah—if the doctor talks to you about “the reminder to take pills” coming and it’s gonna be three beeps...or...one song...something they can look forward to from the doctor...I’d want one song as my “beep”: Bob Marley, Three Little Birds, something can smile about, connect to.

P7, Native Hawaiian/Pacific Islander

Use a sound but it is going to be gospel music, a very popular gospel song. Pretty soon, it is going to get stuck in their head, you want to use a very popular gospel song.

P15, African American

#### Elements Wanted by Native Hawaiian and Pacific Islanders

Several themes were unique to the Native Hawaiian and Pacific Islander community. These included (1) need for a plan, (2) patient partnership and engagement, (3) reduction of medications if possible, and (4) consider level of readiness.

##### Need a Plan

One common sentiment among the Native Hawaiian and Pacific Islander group was the need to have a plan that the patient helps to develop:

Needs to be part of a bigger coordinated plan of care it can become useless...quickly...if no feedback step incorporated with the text...

P9, Native Hawaiian/Pacific Islander

##### Patient Partnership

Gatekeeper-stakeholders also stated that patients need to be active partners in decisions and interventions need to adapt to their needs:

It’s gonna be different for everybody—not one size—one set fits all—need them (Patient) feedback and commitment to whatever is decided with a process in place to make changes if needed. Depends—midmorning always a good start—but needs to be personalized and agreed upon with the patient.

P3, Native Hawaiian/Pacific Islander

##### Reduction of Medications at Start (if Possible)

Several Native Hawaiian and Pacific Islander gatekeeper-stakeholders brought up the idea that patients want to be on fewer medications, if possible:

Patients don’t want more pills—they want less, they want to feel better, they want to try anything before taking more blood pressure pills.

P8, Native Hawaiian/Pacific Islander

#### Elements Wanted by African Americans

Some ideas for a successful intervention that were only mentioned by the African American gatekeeper-stakeholders included (1) having workshops to train, (2) involving the church, (3) incentivizing participation, (4) involvement of family, and (5) quotations from scripture or famous authors or information on hobbies.

##### Workshop

Most African American gatekeeper-stakeholders said having group workshops at the church or community center would be a good way to start the intervention:

The majority of individuals are retirees and they are just sitting at the center to pass the day. They will come to workshops because it will be something new to them, they will participate. They will gain the knowledge of how to go through the cell phone, what to do, how to do. That would be at the top of the agenda, an introductory course.

P12, African American

They come in groups, they are not going to come individually. We need to tell them “let me show you how to do this” and then they will do it.

P13, African American

Workshops (including church workshops) can help improve access and increase knowledge of mobile technology among elderly populations. Have a representative (poster person) describe the benefits of research performed on them.

P12, African American

##### Involvement of Church

African American gatekeeper-stakeholders emphasized the need to involve the church in health improvement efforts:

In the black community, you need to somehow link your workshop to the churches and talk about the importance of using mobile for this purpose. Also, you need to identify those gatekeepers who had a good relation with seniors and also pastors and make them involved with your workshops in churches. You might want to have a primary evaluation about each senior’s knowledge and education background about using cellphone and then have your workshops on different levels.

P15, African American

##### Subsidize/Incentivize

Many African American gatekeeper-stakeholders also mentioned the benefits of financial incentives to participate:

Regarding the teacher of this workshop, I would say that this age population group love church. The senior citizen center and some schools are also could be wonderful to have the workshops. One of the incentives to come is providing food at the workshops. Since most group of this population has fixed income, they would appreciate change in their hands (like $30 is excellent). Also, this age group is the Target and Walmarters. Giving them a gift card [to] Target would be excellent.

P16, African American

##### Involvement of Family

Many of the African American gatekeeper-stakeholders recommended involving the family in delivering the messaging and incorporating their voices and photos:

You could ask them when you have an appointment to bring one of their children or grandchildren in so that they could have a partner, who they’d love to be in touch with, someone that they love. Preferably that person would be comfortable with technology so that they can help with keeping said person on his or her medication schedule.

P17, African American

You also could send the picture of the medication or even a picture of his/her son/daughter...as a reminder to them.

P12, African American

##### Quotes from Scripture/Authors and Information on Hobbies

In addition, many African American gatekeeper-stakeholders suggested incorporating quotations from scripture or famous authors into the messaging:

In their first interview, ask them about their favorite scripture (if they have one). Ask them about their favorite writer and then you can put some sentence from that writer. For instance my favorite activist is Marian Wright Edelman and her favorite motto is “Service is the rent we pay for being. It is the very purpose of life, and not something you do in your spare time.” I would love seeing that every morning. If you want, ask them about their hobby and interest for phrasing your text, you have to make sure they are comfortable with it and know why you are asking this question and how it connects to your study. You need to ask them in a very nice way, such as “We would like you to wake up or to go to rest with a thought that you find interesting or pleasant. What would be the most interesting in having us say to you or send to you?” and then have the list of those kind of things: their hobby, their favorite writer, and their favorite scripture.

P16, African American

Be careful using faith-based statements straight from the Bible. It may feel like an intrusion into their faith. Possibly use statements from nonreligious influential people: Maya Angelou, Martin Luther King, Jr...Start with a phrase of gratitude like “God woke me this morning,” but I do not think it is necessary to get into faith. Have messages come from patients, people that are good at adherence, like why they take their medications every day. There is peer credibility.

P17, African American

### Barriers to Success

In discussing whether a mHealth intervention would be successful, gatekeeper-stakeholders mentioned several barriers to success (Textbox 3). The main themes in this area were (1) there are other reasons for nonadherence (side effects, distrust, and depression), (2) they do not always have their mobile phone with them or do not have it on unless talking to family or in an emergency, and (3) they do not like text messages or need eye-to-eye contact. Several gatekeeper-stakeholders mentioned the fact that there are many reasons patients do not take their medications. This intervention will primarily help those who do not take their medication due to forgetfulness, but does not address other causes of nonadherence.

In addition, the Native Hawaiian and Pacific Islander gatekeeper-stakeholders mentioned some other barriers, including that we need to be specific about medications because they may not be sure which pill we are talking about if we just say “blood pressure medication” because pills are constantly changing. Another issue that was brought up was that the mobile phone keys are small so it may be difficult for participants to text responses. Finally, someone mentioned that it is likely that even if patients become more adherent initially, they may lose interest in the intervention and stop participating. This raises a question about the sustainability of the impact of the intervention.

**Barriers to a successful mHealth intervention by group.**

**Both groups**

- Other reasons for nonadherence (side effects, distrust, depression)
- Do not carry mobile phone or have it off unless talking to family or in an emergency only
- Do not like text messages; need eye-to-eye contact
- Keys are small; text hard to read

**African Americans**

None stated from this group

**Native Hawaiians/Pacific Islanders**

- Not sure which pill is which; pills constantly changing
- Do not want to sound like telemarketing / be annoying
- People may lose interest and stop participating

Here are some of the statements that the gatekeeper-stakeholders made regarding barriers:

Depression could be a barrier to people using medications. People have individualized reasons why they do not adhere to medication.

P17, African American

...but gotta remember people don’t take their medications for a lot of reasons—it’s not just that they forget—so a daily text for those with other reasons might not work. They don’t believe it helps, don’t trust the doctor, don’t want to question the doctor, and just don’t take the pill or don’t even fill the prescription...social barriers in terms of homelessness, money, financial.

P6, Native Hawaiian/Pacific Islander

I need eye-to-eye contact, face-to-face contact. I am not text savvy. If you don’t have time to call them, you can send a prerecorded message to them with a voice of their close friends or relatives (instead of having a real person call).

P15, African American

An African American gatekeeper-stakeholder mentioned the elderly may be scared of new technology:

Overall, I think seniors are scared of technology, but if people that they trust persuade them, then they will make the transition.

P17, African American

In general, even though gatekeeper-stakeholders discussed barriers, they seemed to think that these barriers could be overcome, particularly if the connection, trust, and caring were present.

### Phrasing

When asked what specific phrasing we should use, gatekeeper-stakeholders suggested:

“It’s time again...Mrs/Mr..., it’s time for your morning pill. Just a friendly reminder. It’s time to take your pill. It’s time to take your BP medicine. Even if you feel ok, remember to take your BP medicine. Taking your BP medicine as recommended is important for your health. It’s time to take yours. It’s time for your BP medicine. How about a short walk afterwards if you are able and it’s ok with your doctor. It’s that time again. Remember to take your BP medicine. Keep your BP medicine in a place that helps you remember to take it. It’s time now.”

P11, African American

Good morning. This is just a reminder that it’s time for you to take your blood pressure medicine.” We have to be community-friendly so we have to use their terms. We cannot use “hypertension” we need to say “high blood pressure.” Giving them a directive is offensive. Make it like you are talking to them.

P13, African American

In the morning:

“Good morning, Chauncy, sleepy head. It’s time to take your blood pressure medication.” Tell them the name of the medication because they could take the wrong thing at the wrong time. Tell them any important instruction for taking the medication...Tell them you do have to text back this time—send “yes” or “oops” if you took it or not.

P14, African American

### Cultural Considerations

Cultural considerations varied by group. Among the African American group, the main consideration was lack of trust in the medical system. As a result, many suggested that the messaging should come from someone outside the medical/research community, such as a family member, friends, pastor, or someone else they trusted (Textbox 4), whereas the Native Hawaiian and Pacific Islander group mainly suggested that providers send the messages.

**Who should send the reminders and who should receive them?**

**Who Should Send the Reminder?**

**Both groups**

- Doctor/provider
- Pharmacist
- Patient choice

**African Americans**

- Family member/friend
- Study personnel
- Pastor
- Community liaison
- Peer

**Native Hawaiians/Pacific Islanders**

- Whoever is most engaged and taught them program
- Knows patient and medical history

**Who Else, Besides the Patient, Should Receive the Reminder?**

**Both groups**

- Family/spouse/companion
- Caregiver / senior housing
- Patient choice
- Pharmacist

**African Americans**

- Peer with hypertension
- Doctor

**Native Hawaiians/Pacific Islanders**

None stated by this group

Some older people may think it is rude to use the first name, like it is disrespectful and want to be called by Mrs/Mr and their last name (because they are coming out of serious racism where they called boy and things like that). They might say, “You don’t know me, don’t call me by my name.” But, the younger people, they don’t care. “Good morning Mrs/Mr so and so.” I would go with that, since it’s safe.

P17, African American

*If a voice doesn’t sound African American...I don’t trust it. If it sounds European, I’m like those people are talking again...It has to be culturally appropriate.* (P18, African American)

People of color in general still have a mistrust of the medical system. Most people score moderate to extreme distrust of the medical system and they are around 40; that mistrust might increase as they get older. Something culturally important must increase trust in people who are doing this particular project. Remove the mistrusted person and replace with the family member or church leader. Community or family liaison is critical to patients trusting in the project.

P19, African American

For the Native Hawaiian and Pacific Islander group, trust of the system did not appear to be as big an issue. Most, however, expressed the need to deliver the intervention with warmth, caring, and aloha from someone with whom they have a connection:

I think warmth—aloha—so they know that we care for them about how they’re doing and not just grabbing them to do something—it has to matter and what they do has to matter—a text can’t be cultural—it has to...come from the sender that it matters that they matter.

P2, Native Hawaiian/Pacific Islander

Yeah important to always show aloha, we do at the clinic always and that can work in the app too—personalized to what’s important to them we can personalize it together.

P5, Native Hawaiian/Pacific Islander

Although both the African American and Native Hawaiian and Pacific Islander groups expressed trust as an issue, it seemed to be a more important consideration in the African American community. The Native Hawaiian and Pacific Islander gatekeeper-stakeholders emphasized the need for aloha and warmth.

## Discussion

Overall, the response from gatekeeper-stakeholders was very positive toward the potential for a mHealth intervention to improve adherence to antihypertensives among elderly adults. This acceptability of mHealth is consistent with a prior study of a culturally diverse low-income population at two primary care clinics in California [29]. In this study, 86% of respondents said they were interested in using mHealth to improve their health. Our study differs in that we focus on medication adherence and acceptance of mHealth in elderly African Americans and Native Hawaiian and Pacific Islanders.

A number of similar themes regarding what would be needed to make this intervention successful emerged from our two groups of African American and Native Hawaiian and Pacific Islander gatekeeper-stakeholders). First was the need to teach participants both about the importance of adherence to antihypertensive medications and the use of mobile phones for messaging. Second, gatekeeper-stakeholders in both communities expressed a strong need for participant access to ongoing “technical support” and “checking in” to make sure everything was going okay. Therefore, it is important that interventional studies that target older adults and intend to use mobile phones provide culturally appropriate trainings for these populations. We were repeatedly reminded by our participants that older adults are very interested in and excited about learning how to effectively operate their mobile phone. They also advised that such trainings should be provided in a friendly environment, adjusted to their level of understating and needs, and be delivered by a peer educator. In addition, they noted that trainings should continue during the course of interventions and repeated frequently to enhance their usage.

Third, almost all gatekeeper-stakeholders mentioned the need to personalize the intervention to the needs of the participant in terms of frequency of text messages, who should be sending the text message, what it should contain (eg, quotes from scripture). Fourth, the messaging needs to come from someone the participant trusts and with whom they have a connection. Fifth, the messaging needs be caring and warm, not cold and impersonal. Finally, short, simple, and direct, but personalized, is the best type of messaging.

However, African American and Native Hawaiian and Pacific Islander gatekeeper-stakeholders did tend to differ on some thoughts regarding the intervention. For instance, there was a strong sentiment among the African American group that the church be involved and that the intervention begin with group workshops, whereas the Native Hawaiian and Pacific Islander group seemed to believe that the teaching could occur on a one-to-one basis with the health care provider (eg, physician, pharmacist, community health worker). Along the same lines, the African American group was also much more likely to recommend including verse from scripture or quotes from famous authors in the messaging, as well as incorporating tips on hobbies and recipes. Moreover, a distrust of the medical system was more commonly expressed among the African American gatekeeper-stakeholders, leading to the suggestion that the messaging originate from someone outside the medical system, such as a family member, pastor, or peer.

In conclusion, our study provides the first qualitative assessment of perspectives on use of mHealth technology to improve medication adherence among elderly African Americans and Native Hawaiian and Pacific Islanders. Successful interventions need to be personalized to meet patient needs, come from a place of caring from a trusted person, and be short and simple to understand.
